# Heterogeneity of juvenile depression with different risks of developing psychosis according to immunological blood parameters

**DOI:** 10.1192/j.eurpsy.2025.2038

**Published:** 2025-08-26

**Authors:** S. A. Zozulya, T. I. Shishkovskaya, I. N. Otman, I. V. Oleichik, Z. V. Sarmanova, P. A. Baranov, L. V. Androsova

**Affiliations:** 1 FSBSI “Mental Health Research Centre”, Moscow, Russian Federation

## Abstract

**Introduction:**

One of the mechanisms underlying the pathogenesis of mental disorders, including endogenous depression, is systemic inflammation. It is of interest to study the immunological aspects of the early stages of endogenous disorders and identify subgroups of patients with immunotypes that characterize a high risk of developing the first psychotic episode.

**Objectives:**

Comparative analysis of the spectrum of inflammatory markers in patients with a juvenile depression with high and low risk of developing psychosis.

**Methods:**

The study involved 98 women 16 to 25 years (20.9 ± 5.14 years) with depression within the framework of various nosologies (F31.3-4; F33.0-1; F60.0-9; F21.3-4; F20.01-2; F25.1). Two groups of patients without a history of psychosis were identified: group 1 (n = 47) - without symptoms of psychosis risk, group 2 (n = 51) - with depression associated with psychopathological symptoms of psychosis risk. The control group consisted of 42 healthy women of the corresponding age. The severity of depressive symptoms was assessed using the HDRS-21, the severity of negative and positive symptoms was determined using the SANS and SAPS. In group 2, the severity of attenuated positive symptoms was determined using the SOPS. The activity of the leukocyte elastase (LE) and α1-proteinase inhibitor (α1-PI), as well as the level of autoantibodies (AB) to S100B and MBP, were determined in plasma.

**Results:**

The groups were characterized by a statistically significant increase in both LE and α1-PI (p<0.05), and the level of AB compared to the control (p<0.05), but no significant differences were found. In group 1, clinical and biological correlations were found between LE activity and the total score on the SANS (r=0,44, p=0.002). In group 2, a negative correlation was found between LE activity and the age of onset of the disease (r=-0.3, p=0.046).

The clustering of patients by LE activity and their distribution by immunological groups showed that 29.4% and 27.5% of patients in groups 1 and 2, respectively, were characterized by a high level of inflammatory markers and the absence of an autoimmune component to neuroantigens, which is a sign of a more favorable course of the pathological process. On the contrary, 70.6% and 72.5% of patients in groups 1 and 2, respectively, were characterized by the type of inflammatory response associated with an increase in the level of AB and varying degrees of insufficiency of the functional activity of neutrophils, which is considered an unfavorable factor that aggravates the course of the disease.

**Conclusions:**

Comparison of the spectrum of inflammatory markers in juvenile depression with different risk of developing psychosis indicate their significant immunological heterogeneity. The immunotype characterized by a high level of AB and insufficient LE activity can presumably be considered as a predictor of the risk of developing psychosis.

**Disclosure of Interest:**

None Declared

